# Beware Postpartum Shortness of Breath

**DOI:** 10.12669/pjms.315.8060

**Published:** 2015

**Authors:** Guleser Akpinar, Afsin Ipekci, Bedia Gulen, Ibrahim Ikizceli

**Affiliations:** 1 Guleser Akpinar, MD, Department of Emergency Medicine, Sisli Hamidiye Etfal Training & Research Hospital, Istanbul, Turkey; 2 Afsin Ipekci, MD, Department of Emergency Medicine, Cerrahpasa Faculty of Medicine, Istanbul, Turkey; 3 Bedia Gulen, MD, Department of Emergency Medicine, Bezmialem University School of Medicine, Istanbul, Turkey; 4 Ibrahim Ikizceli, MD, Professor, Department of Emergency Medicine, Sisli Hamidiye Etfal Education & Training Hospital, Istanbul, Turkey

**Keywords:** Cardiomyopathy, Heart failure, Peripartum cardiomyopathy

## Abstract

Peripartum cardiomyopathy (PPCM) is one of the potentially life-threatening complications of pregnancy. We report a case of a 36-year-old female patient who presented with shortness of breath, swelling of feet after giving birth to triplets, and her tests revealed that left ventricle is dilated with its diameter on the borderline and she had EF 35% with advanced systolic dysfunction. Anterior wall and septum were severely hypokinetic. In the presence of these findings, the patient was evaluated as PPCM. PPCM must be considered in the differential diagnosis of a patient presenting with shortness of breath and swelling of feet, which are also common in pregnancy.

## INTRODUCTION

Peripartum cardiomyopathy (PPCM) is one of the potentially life-threatening complications of pregnancy, the underlying reason for which is unknown. This form of dilated cardiomyopathy causes congestive heart failure in the later months of pregnancy or in the first 5 months after birth. Whether to define this profile as an entity specific to pregnancy or not is still a subject of discussion.^1^ In many cases classified as idiopathic, after careful examination, various underlying causes, such as chronic hypertension, mitral stenosis unnoticed, obesity, viral myocarditis, preeclampsia, anemia, infection can be detected.^2^

Clinical course of the disease may vary between spontaneous recovery of ventricular functions and the disease becoming refractory and development of a need for a heart transplant. Early diagnosis, treatment, and clinician being familiar with this disease are the basic factors that improve the prognosis.^3^ In our case, we aimed to present the diagnosis and treatment of PPCM.

## CASE REPORT

**Fig.1:**
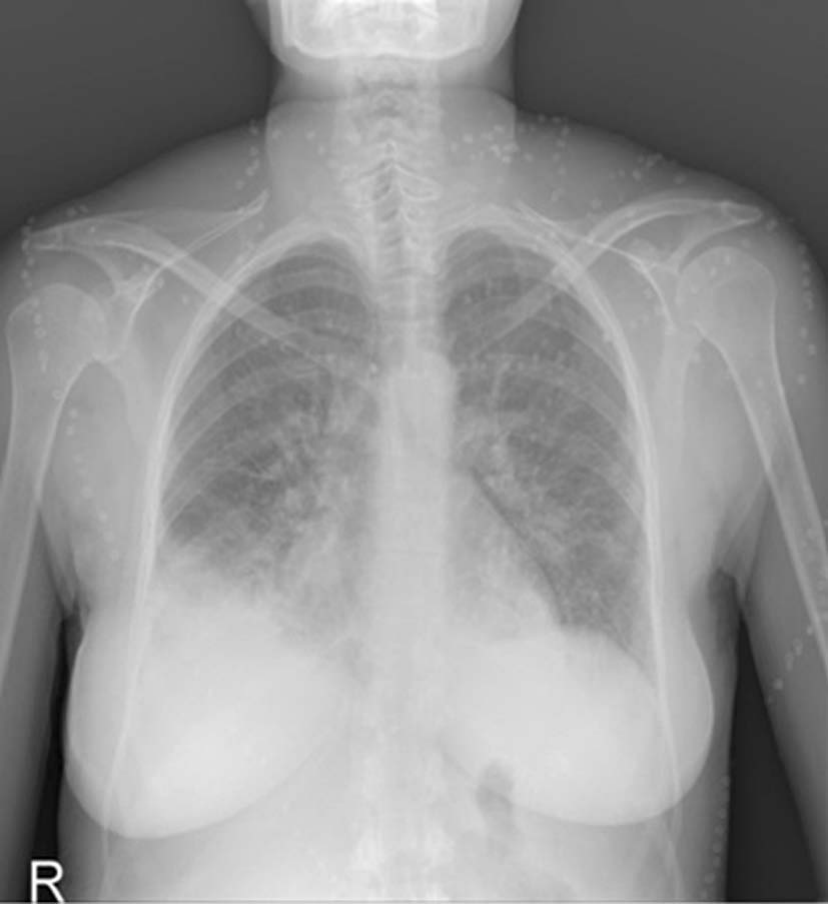
Anteroposterior chest radiography in a 36-year-old woman demonstrates right pleural effusion.

**Fig.2:**
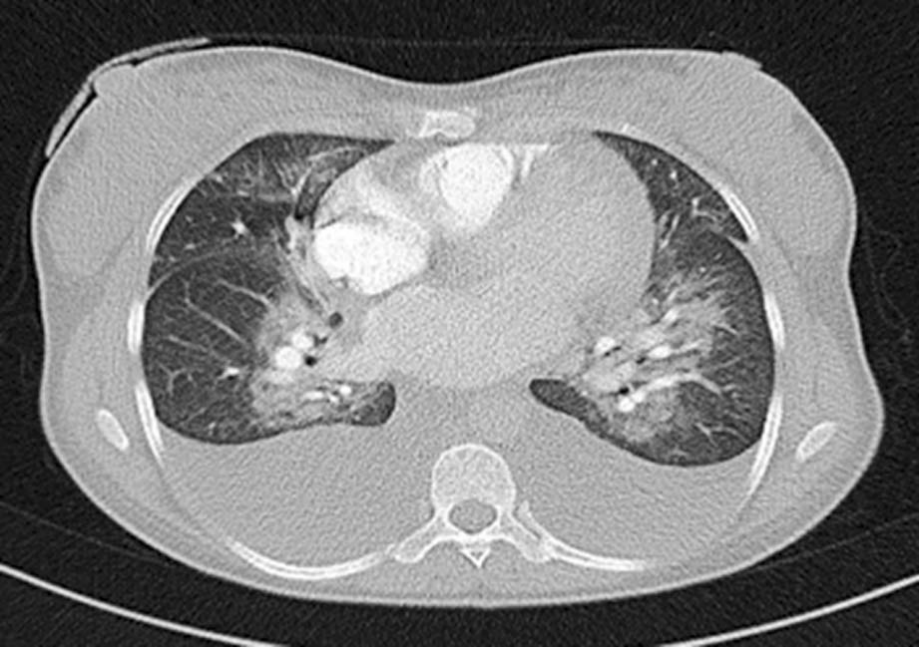
Computed tomography scan of the chest demonstrates bilateral pleural effusion.

A 36-year-old female patient presented to our department 5 days after giving birth to girl triplets at week 35 by cesarean section with shortness of breath, swelling of feet. The patient’s history includes normal vaginal deliveries in 2001 and 2006, and the patient had two healthy girls. She had no history of known hypertension, preeclampsia or cardiac disease and developed no complications in the early postoperative period. In her physical examination, her general state was mediocre, she was pale and had dyspnea and orthopnea. She had +2 positive pitting edema in bilateral lower extremities. In her lung auscultation, her bilateral sounds were reduced and bilateral rales were present. In her cardiovascular system examination, S1 + S2 were rhythmic and tachycardic. Her vital signs were as follows: BP:120/70 mmHg, pulse:110/min, temperature:37 Cº, saturation O2: 90%. For differential diagnosis, the patient had hemogram, biochemistry, blood gas tests done and underwent an EKG. Her test results were as follows: arterial blood gas: PH:7.39, PCO2:26.6, PO2:49.5, SO2:83.5, CRP:23.26 mg/L, White blood cell :21.79 K / uL (4-11 K / uL), Hemoglobin:12 g / dL (13-17.5 g / dL), Platelets:647 K / uL (150 – 400 K / uL), Aspartate transaminase: 41 U/L (15 – 32 U/L), Alanine Transaminase: 20,9 U/L (10 – 33 U/L), LDH: 370 U/L (135-214 U/L), Troponin: 0,354 ng/mL.(<0.12 ng/mL) CKMB:24,54 ng/mL (0-4.88 ng/mL). Anteroposterior chest X-ray and tomography of the chest showed bilateral pleural effusion (Fig. 1 and 2). A 12-lead EKG revealed normal sinus rhythm; left bundle branch block and sinus tachycardia were present. Echocardiogram (ECHO) showed that left ventricle was dilated with its diameters on the borderline and there was EF 35% severe systolic dysfunction. Anterior wall and septum were severely hypokinetic.

In the presence of these findings, the patient was evaluated as PPCM and acute coronary syndrome in emergency department and transferred to the intensive care unit of cardiology. The patient was started on Lasix 3*20mg IV, Aldactone 25 mg tablets, Beloc 50 mg tablets. Her follow-up test results were as follows: CRP:13,10 mg/L, White blood cell :7,3 K / uL (4-11 K / uL), Aspartate transaminase: 26 U/L (15 – 32 U/L), Troponin:0,100 ng/mL.(<0.12 ng/mL) CKMB:2.48 ng/mL (0-4.88 ng/mL). She was discharged with the diagnosis of PPCM and low-salt diet, digoxin, diuretics, and vasodilator agents treatment was started as the conventional treatment of heart failure.

## DISCUSSION

Pregnancy leads to a variety of anatomical and physiological changes. The resulting changes greatly affect pulmonary and cardiovascular systems. Particularly in the case of those with cardiopulmonary disease before pregnancy, pregnancy causes exacerbation of underlying disease, while certain conditions only develop during pregnancy or are specific to pregnancy (amniotic fluid embolism, pulmonary edema developing with tocolytic treatment, etc.).

PPCM is a diagnosis made by excluding other causes, when another cause of heart failure is not detected. Although the left ventricle is not dilated, EF almost always goes down below 45%. The incidence of PPCM is 1/3000 - 1/4000 and PPCM accounts for approximately 4% of maternal deaths.^4^ Predisposing factors include multiple pregnancy, multiple birth, family history, ethnicity, smoking, diabetes, hypertension, preeclampsia, malnutrition, having children at an advanced age, pregnancy in the second decade of life, and long-term beta-blocker use. Etiology of PPCM covers cardiotropic viruses, autoimmune disorders, toxins causing immune system dysfunction, abnormal serum relaxin levels, selenium deficiency, the presence of pro-inflammatory cytokines, antibodies giving high titers of abnormal response to cardiac tissue and underlying myocarditis.^5^,^6^ In our case, the patient had a spontaneous multiple pregnancy, and EF was 35%.

PPCM patients consult a doctor with signs and symptoms related to congestive heart failure. They may present with complaints of shortness of breath, cough, tachypnea, and cyanosis. PPCM’s physical examination findings include edema, rales, jugular vein distention, third and fourth heart sounds. The most important finding is cardiomegaly. In differential diagnosis, pulmonary thromboembolism, one of peripartum complications, pneumonia, amniotic fluid embolism and asthma should be considered.^7^

Clinical diagnostic criteria for the disease were determined by Demakis et al. in 1971 as follows:^8^

1. Development of heart failure in the last month of pregnancy or within the first 5 months after delivery.
2. Absence of other causes of heart failure.
3. Absence of a defined heart disease before the last month of pregnancy.

In 1999, left ventricular dysfunction manifesting itself in ECHO by a decrease in ejection fraction was added to these criteria.^9^ In our case, the patient had no previous history of cardiac disease, the disease developed in postpartum period, no reason could be identified for heart failure and she had matching ECHO findings so she was diagnosed with PPCM and transferred to ICU of cardiology department.

PPCM treatment is carried out in the same manner as conventional heart failure therapy; oxygen supplementation, a low-salt diet, diuretics, digital and vasodilator agents are the main instruments. PPCM may cause congestive heart failure, increased atrioventricular arrhythmias, thromboembolism, and sudden death.

Patients with postpartum cardiomyopathy are at high risk for development of thrombosis and thromboembolism due to pregnancy-related hypercoagulation as well as blood stasis associated with severe systolic dysfunction. Therefore, adding anticoagulant therapy to standard heart failure therapy should be considered.

A consecutive pregnancy carries a recurrence risk of 30-50% for PPCM. Unless EF becomes normal, a subsequent pregnancy should be discouraged. Even if EF has become normal, the patient should be given consulting services due to recurrence risk of the disease in a new pregnancy.^10^

Although a mortality rate of 50% was established for the disease in the 1950s, today, Duran et al. reported a mortality rate of 30.3%, a heart transplantation rate of 6.1% and a persistent left ventricular dysfunction rate of 39.4%.^11^

## CONCLUSION

PPCM is a disease with which the patients are diagnosed by excluding other possible diagnoses. It is a pregnancy complication with unknown etiology and lethal potential. If the clinician is familiar with the disease, the respective patient can be diagnosed faster, treated appropriately and prognosis will be the best.

## Authors’ Controbutions

***Akpinar G, Ikizceli I, Gulen B:*** Substantial contributions to the conception of the work. ***Akpinar G, Gulen B:*** Drafting the work and revising it critically for important intellectual content. ***Akpinar G, Ipekci A, Ikizceli I:*** Final approval of the version to be published. ***Akpinar G, Ipekci A:*** Agreement to be accountable for all aspects of the work in ensuring that questions related to the accuracy or integrity of any part of the work are appropriately investigated and resolved.
